# Warts

**Published:** 1886-11

**Authors:** 


Warts.—Dr. D. C. Platt recommends the following application to remove warts:

| | | | |
|---|---|---|---|
| ℞ | Argenti Nitratis, - - - - | ʒ i. | |
| | Acid-Nitromuriatic, - - - - | ℥ i. | M. |

Sig.:—Apply once a day.—*New England Med. Monthly.*

